# Regeneration of non-chimeric plants from DNA-free edited grapevine protoplasts

**DOI:** 10.3389/fpls.2022.1078931

**Published:** 2022-12-01

**Authors:** Simone Scintilla, Umberto Salvagnin, Lisa Giacomelli, Tieme Zeilmaker, Mickael A. Malnoy, Jeroen Rouppe van der Voort, Claudio Moser

**Affiliations:** ^1^ Centro Ricerca ed Innovazione, Fondazione E. Mach. Via E. Mach 1, San Michele all’Adige, Trento, Italy; ^2^ Consorzio Innovazione Vite (CIVIT), Trento, TN, Italy; ^3^ Scienza Biotechnologies BV., Enkhuizen, Netherlands

**Keywords:** grapevine, protoplast, RNPs, genome editing, CRISPR/Cas9

## Abstract

The application of New Breeding Techniques (NBTs) in *Vitis vinifera* is highly desirable to introduce valuable traits while preserving the genotype of the elite cultivars. However, a broad application of NBTs through standard DNA-based transformation is poorly accepted by public opinion and law regulations in Europe and other countries due to the stable integration of exogenous DNA, which leads to transgenic plants possibly affected by chimerism. A single-cell based approach, coupled with a DNA-free transfection of the CRISPR/Cas editing machinery, constitutes a powerful tool to overcome these problems and maintain the original genetic make-up in the whole organism. We here describe a successful single-cell based, DNA-free methodology to obtain edited grapevine plants, regenerated from protoplasts isolated from embryogenic callus of two table grapevine varieties (*V. vinifera* cv. Crimson seedless and Sugraone). The regenerated, non-chimeric plants were edited on the downy- and powdery-mildew susceptibility genes, *VviDMR6* and *VviMlo6* respectively, either as single or double mutants.

## Introduction

Genome editing technology allows to modify cellular DNA with a high level of precision. The advent of CRISPR/Cas (*Clustered Regularly Interspaced Short Palindromic Repeats/*Cas) technology has widely extended the application fields of genome editing. Based on the recognition of the DNA-editing site through a complementary RNA sequence followed by a nuclease-mediated double strand break, this system makes possible small insertions and deletions or modification of one single nucleotide (Chen et al., 2019). The CRISPR/Cas components can be introduced inside a cell either in the form of nucleic acids (i.e. DNA or mRNA coding for the entire system), or in the form of ribonucleoprotein (RNP) complex. However, while DNA might integrate into the genome and mRNA is affected by its intrinsic instability, direct cellular delivery of RNPs provides a robust methodology leading to a specific and minimal mutation with no trace of exogenous DNA (Woo et al., 2015). With this perspective, the interest on the application of CRISPR/Cas technology to plants is rapidly raising, since improved crops obtained with this strategy would potentially benefit of a better acceptance by consumers with respect to the classic transgenic crops (Entine et al., 2021).

Three main strategies have been proposed so far to deliver the CRISPR/Cas system into plant cells. 1) The use of engineered binary vectors (Ren et al., 2016) mediated by *Agrobacterium tumefaciens*, which has the natural ability to overcome the plant cell wall. However, this strategy employs an exogenous DNA source which gets integrated in the cellular DNA upon transformation (Gelvin, 2017). In the case of woody plants, the removal of the exogenous DNA can only be achieved by outcrossing, with consequent (and frequently unwanted) change of the plant original genetic make-up. Alternatively, the application of DNA molecular excision (Pompili et al., 2020) by means of currently available protocols would not lead to a full excision of the T-DNA, with the remaining traces of exogenous DNA being sufficient to consider these plant as GMOs in many countries, according to the existing regulation (Dima and Inzé, 2021). 2) The use of particle bombardment (Ozyigit and Yucebilgili Kurtoglu, 2020), in which nanoparticle bullets loaded with CRISPR/Cas components are shot onto plant tissues thereby overpassing the cell wall barrier and releasing the editing machinery directly inside the cell. However, the biological material must be released inside the nucleus for a successful delivery and various physical parameters severely affect the overall efficiency of the technique. In addition, as not all the cells would be hit by the nanoparticles, the downstream regeneration process may produce chimeric plants. 3) The use of plant protoplasts, where the cell wall is temporarily removed through enzymatic digestion. Once obtained, protoplasts can be easily transfected by means of classic methodologies such as polyethylene glycol (PEG)-mediated transfection or electroporation (Reed and Bargmann, 2021). Although intrinsic difficulties bound to regenerate an entire organism might arise, depending on the nature of the tissues as well as on the species considered (Reed and Bargmann, 2021), a strategy based on regeneration of protoplasts might be highly beneficial. It would not only allow for a DNA-free editing approach, but, since plants are regenerated from single cells, it would also provide the advantage of avoiding chimerism, thus ensuring the desired genetic homogeneity. The single cell strategy has been tested on many crops such as banana, lettuce, chicory, and many species of Brassicaceae (Woo et al., 2015; Cankar et al., 2021; Reed and Bargmann, 2021). Applied to grapevine, it would allow to preserve the genotype of elite cultivars while still resulting in precise genetic modifications. Despite woody-plant protoplasts are relatively easy to obtain from several tissues, their editing efficiency is generally low and the regeneration process is frequently unsuccessful (Malnoy et al., 2016; Osakabe et al., 2018). Indeed there are very few reports in the scientific literature about successful regeneration of whole plants from grapevine (both *vinifera* and *Vitis* hybrids) protoplasts (Reustle et al., 1995; Zhu et al., 1997; Bertini et al., 2019; Tricoli, 2019) and, to the best of our knowledge, regeneration of whole grapevine plants from DNA-free edited protoplasts has been reported only once and very recently (Najafi et al., 2022).

The present work provides a methodology to efficiently i) obtain protoplasts from grapevine embryogenic callus; ii) edit the protoplasts at target genes through CRISPR/Cas9, and iii) regenerate fully edited grapevine plants.

**Figure 1:**
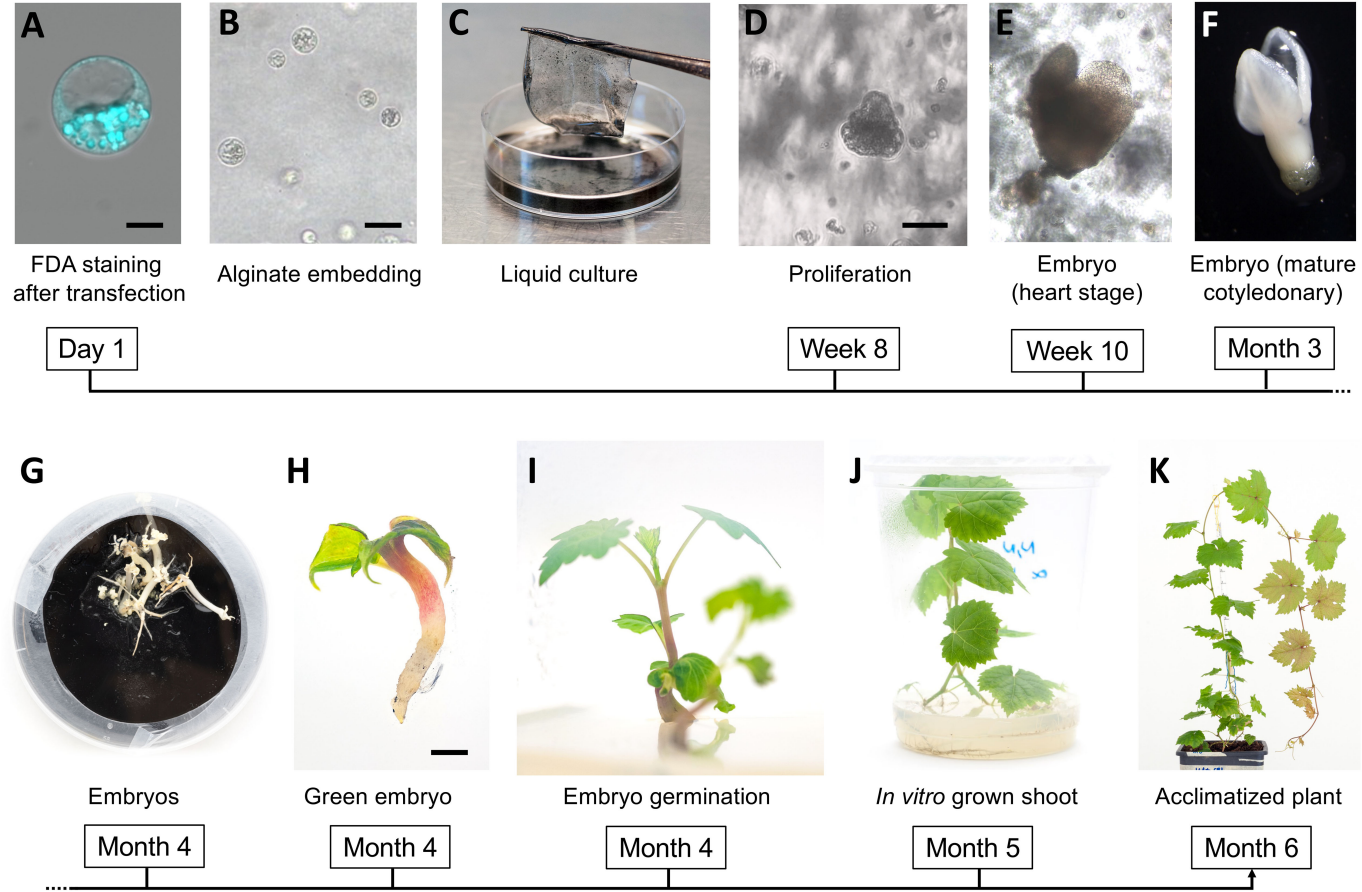
Regeneration of grapevine plants from protoplasts. FDA-staining of a viable protoplast from the embryogenic callus digestion mixture after transformation **(A)**; protoplast embedded in the alginate disc **(B)**; alginate disc set to float in the NN-based medium **(C)**; aggregate of proliferating cells **(D)** and first embryos at heart stage **(E)**; embryo at mature cotiledonary stage **(F)** and final stage **(G)** on solid GS1CA medium; green embryo after exposure to light **(H)**; emerging shoot during embryo germination **(I)**; in vitro plant at 40 days after exposure to light **(J)**; acclimatized plant at one month after potting **(K)**. Black magnification bars correspond to 5 μm, 50 μm, 100 μm, and 2mm in **(A, B, D, H)**, respectively.

In this study, the single cell approach was successfully applied (**Figure 1**) to embryogenic calli obtained from two table grapevine varieties, *V. vinifera* L. cv. Crimson seedless and Sugraone. Two plant genes were chosen as candidate targets for CRISPR/Cas knockout experiments: *VviDMR6* (*Downy Mildew Resistant* 6) and *VviMLO6 (Mildew Locus O* 6). These genes are likely involved in susceptibility to two of the most severe grapevine diseases, i.e. downy mildew (DM) and powdery mildew (PM), respectively. Thus, a successful genome editing strategy for creating novel grape genetics may have large impact on grape disease management.

## Material and methods

### Embryogenic callus production and maintenance

Embryogenic calli of Crimson seedless and Sugraone were initiated from immature inflorescences collected from a vineyard in San Michele all’Adige (Trento, Italy). Flowers were surface sterilized for 20 minutes in diluted bleach (3% active hypochlorite) and rinsed with sterile distilled water for other 20 minutes before placing them in the fridge. After two to four days, anthers with filaments and ovaries were cut under a stereomicroscope and processed as described in (Martinelli et al., 2001).

### Isolation of protoplasts

Protoplasts were isolated from 1 g of embryogenic callus of either Sugraone or Crimson S. in 13 ml of enzymatic mixture composed of 1% (w/v) cellulase Onozuka R-10 and 0.3% (w/v) macerozyme R-10 (Duchefa Biochemie, Haarlem, The Netherlands) plus 0.2% (w/v) hemicellulase (Merck KGaA, Darmstad, Germany) dissolved in Gamborg B5 including vitamins (Duchefa Biochemie, Haarlem, The Netherlands) and 0.45 M mannitol (**Table 1**), in sterile conditions. The suspension was mixed on a tilt shaker at 25°C for 16 hours in the dark, and then filtered through a 60 μm nylon sieve (Millipore, Burlington, MA, U.S.A). Protoplasts were collected by centrifugation at 80 g for 4 minutes without brake, washed in MMG solution (**Table 1**), and further purified on a 16% w/v sucrose cushion by centrifugation (90 g, 4 minutes, no brake). Protoplasts were then checked for plasma membrane integrity through FDA staining as described in literature (Huang et al., 1986). Briefly, a 50X stock solution of FDA in acetone was prepared at 5 mg ml^-1^, and was then added to protoplast suspension in MMG and incubated for 5 minutes before observing at the microscope.

**Table 1:** List of solutions/buffers required to perform protoplast isolation, transfection, and regeneration with the respective composition.

Name/Abbreviation	Composition
W5	2 mM MES · H_2_O, 154 mM NaCl, 125 mM CaCl_2_ · 2H_2_O, 5 mM KCl, pH 5.7
MMG	4 mM MES · H_2_O, 0.4 M mannitol, 15 mM MgCl_2_, pH 5.7
WI	4 mM MES · H_2_O, 0.5 M mannitol, 20 mM KCl, pH 5.7
PEG-Calcium	0.2 M mannitol, 100 mM CaCl_2_ · 2H_2_O, 40% (w/v) PEG4000
B5 solution for callus digestion	Gamborg’s B5 salts including vitamins, 0.45M mannitol, 1% (w/v) Cellulase Onozuka R-10, 0.2% (w/v) Hemicellulase, 0.3% (w/v) Macerozyme R-10, pH 5.7
Sucrose solution	16% (w/v) sucrose, pH 5.7
Alginate solution	0.5M mannitol, 1.6% (w/v) sodium alginate
Calcium-agar	0.4 M mannitol, 50 mM mM CaCl_2_ · 2H_2_O, 1.4% (w/v) plant agar, pH 5.7
Media	
Nitsch and Nitsch based liquid medium for protoplast culture (NNp)	Nitsch and Nitsch salts including vitamins, 88mM sucrose, 300mM glucose, 0.1% (w/v) activated charcoal, 0.93 μM kinetin, 2.22 μM 6-BAP, 10.7 μM NAA, pH 5.7
Solid medium for embryo development (GS1CA)	Nitsch and Nitsch salts including vitamins, 132mM sucrose, 300 μM glutathione, 0.25% (w/v) activated charcoal, 2.22 μM BAP, 5μM NOA, 5.7 μM IAA, 0.42% gelrite™, pH 6.2
Nitsch and Nitsch solid medium for plant growth and propagation	Nitsch and Nitsch salts including vitamins, 66mM sucrose, 0.67% (w/v) plant agar, pH 5.75

### Transformation of protoplasts

Plasmid transformation was carried out as described elsewhere (Yoo et al., 2007). Briefly, 2.5 x 10^5^ cells were added with 20 μg of pKGWFS7 vector (Karimi et al., 2002) in which a CaMV35S promoter was cloned, and gently mixed with 250 μl of freshly prepared PEG-calcium solution (**Table 1**). After washing with WI solution (**Table 2**), cells were embedded in droplets of Nitsch cultivation medium (**Table 1**) + 1% Low Melting Agarose PPC (Duchefa Biochemie, Haarlem, The Netherlands), and cultured in the same liquid medium for up to 3 days. At specific time points, droplets were mounted on a glass slide for microscope imaging and checked for the presence of fluorescence. For RNPs transient transfection, the same number of protoplasts was transfected with RNP complex composed by 40 μg of Cas9 protein (Thermo Fisher Scientific, Waltham, MA, USA) and 40 μg of sgRNA (Merck) targeting either *VviDMR6* (Vitvi13g01119, guide: GGAGGATTGGAGGGCCACTC) or *VviMLO6* (Vitvi13g00579, guide: GCCTACTTGGGCTGTTGCAG). For transformation with Cas9-GFP (Merck, Rahway, NJ, USA) the same conditions were applied, except that RNPs were preassembled with 130 pmol of Cas9-GFP and 1.5 nmol sgRNA targeting *VviDMR6* according to the manufacturer’s instructions (**Table 2**). When protoplasts were used for sequencing, they were maintained in the liquid culture medium for 48 h, then harvested, and their genomic DNA was isolated with classic CTAB extraction.

**Table 2:** List of reagents required to perform protoplast isolation, transfection, and regeneration.

Name/Abbreviation	Origin
Plant agar	Duchefa Biochemie Cat. P1001
Low melting agarose PPC (LMPA)	Duchefa Biochemie Cat. L1204
Gelrite™	Duchefa Biochemie Cat. G1101
MES monohydrate	Duchefa Biochemie Cat. M1503
Active charcoal	Duchefa Biochemie Cat. C1302
Sucrose	Duchefa Biochemie Cat. S0809
Glucose monohydrate	Duchefa Biochemie Cat. G0802
Nitsch medium with vitamins	Duchefa Biochemie Cat. N0224
Gamborg’s B5 medium including vitamins	Duchefa Biochemie Cat. G0210
Cellulase Onozuka R-10	Duchefa Biochemie Cat. C8001
Macerozyme R-10	Duchefa Biochemie Cat. M8002
Hemicellualse from *A. niger*	Sigma-Aldrich Cat. H2125
6-Benzylaminopurine (6-BAP)	Duchefa Biochemie Cat. B0904
Kinetin	Duchefa Biochemie Cat. K0905
α-Naphtalene Acetic Acid	Duchefa Biochemie Cat. N0903
β-Naphtoxyacetic Acid	Duchefa Biochemie Cat. N0912
Indole-3-acetic acid (IAA)	Duchefa Biochemie Cat. I0901
Polyethylene glycol (PEG) 4000	Sigma-Aldrich Cat. 8.07490
Cas9 protein	ThermoFisher™ Cat. A36499
Single-guide RNA (sgRNA)	Merck Custom gRNA
Cas9-GFP protein	Sigma-Aldrich Cat. CAS9GFPPRO
Fluorescein diacetate (FDA)	Sigma-Aldrich Cat. F7378

### Microscopy imaging

For the calculation of transient plasmid transformation efficiency, ten random microscopy fields from two glass slides were analyzed, and the number of fluorescent cells was recorded. Protoplast imaging was carried out using a Leica DMi8 laser scanning confocal microscope (Leica Microsystems, Wetzlar, Germany) at a magnification of 200x. Both FDA and GFP were excited at 488 nm and detected in the 510-560 nm range.

### Protoplasts cell culture and regeneration of plants

Either wild-type or edited protoplasts were embedded in alginate discs as described in literature (Cankar et al., 2021) with some modifications: after their transformation, protoplasts were suspended in WI solution (**Table 1**) at a density of 2 × 10^5^ cells/mL, and then gently mixed in an equal volume of alginate solution (**Table 1**). Then, 1 mL of the resulting suspension was solidified on calcium-agar plates (**Table 1**) by leaving them for 1h at room temperature. To stimulate the formation of micro colonies, alginate discs were cultured on a Nitsch-based liquid medium (**Table 1**) at 24°C in darkness, changing weekly the culturing medium. After 2 weeks, the glucose concentration was progressively diminished by 25% each week, till no glucose was present in the regenerative culture medium after 4 further weeks. The disks were then transferred onto solid GS1CA culture medium (Franks et al., 1998) enriched with 300 μM glutathione (**Table 1**) till formation of embryos, which were transferred onto Nitsch and Nitsch solid medium (**Table 1**) and let regenerate to plants by keeping them at 24°C and a 16/8 light/dark photoperiod (80-100 μmol m^-2^ s^-1^),.

### Sequencing to detect gene editing

The targeted regions of *VviDMR6* and *VviMLO6* were amplified by PCR (Phusion DNA Polymerase, Thermo Fisher Scientific) following the manufacturer’s protocol to obtain a 758 bp amplicon (DMR6 for 5’-TCCCTTTTCCTTCTTTTTGG-3’ and DMR6 rev 5’-AAAATGATGCGGGAGGA CAT-3) and a 368 bp amplicon (MLO6 for 5’- GAGCACCAGCAGAAAAGGGA-3’ and MLO6 rev 5’- AGGAAGGAAATACACGCCATCA-3’) respectively. Sanger sequencing was performed, and the chromatograms were analyzed with SnapGene Viewer 6.0.2 (SnapGene software from Insightful Science, available at snapgene.com). The same regions were analyzed by deep sequencing using an Illumina MiSeq (PE300) platform (Illumina, San Diego, CA, USA) using the MiSeq Control Software 2.0.5. Shorter amplicons were generated with primers (iDMR6 for 5’-GGTTGTCTACCAGTTTCAATGTCA-3’/iDMR6 rev 5’- TGAAGCATGAAAAAGTGTTGTACT-3’; iMLO6 for 5’- AGGGACTTTGATCCATGGCTG-3’/iMLO6 rev 5’- AAGCAGCCTTACCG ATCCAA -3’) containing 5’-overhang adapters to generate the Illumina libraries. The resulting raw pair-end reads were analyzed with the online tool CRISPResso2 [https://crispresso.pinellolab.partners.org/; (Clement et al., 2019)] with default parameters. Editing efficiencies were calculated as the percentage of plants with signs of editing (both mono- and bi-allelic) on the total number of plants.

### Protocol overview

A step-by-step version of the protocol is available in **Supplementary Table 1**.

## Results and discussion

### Protoplast isolation and transient transfection

**Figure 2:**
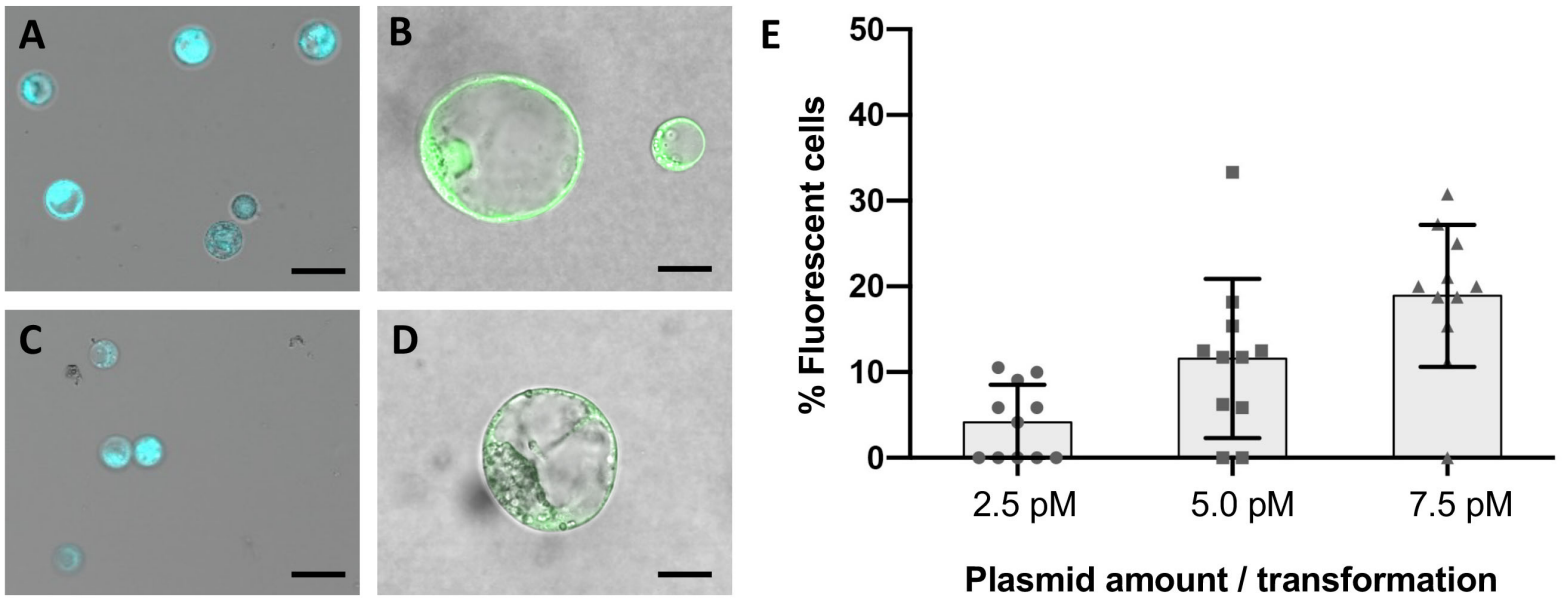
Fluorescence microscopy on wild-type and transformed protoplasts from embryogenic calli. Plasma membrane integrity and cell vitality was assayed with FDA staining in protoplasts suspensions of Crimson s. **(A)** and Sugraone **(C)**. Aliquots of each culture were transformed with a plasmid containing a 35S::GFP expression cassette and embedded in droplets of low melting point agarose for short-term culturing. GFP fluorescence was detected in Crimson s. **(B)** and Sugraone **(D)** samples 24 hours after transformation. Black bars represent 50 μm and 10 μm respectively in a-c and b-d. Plot representing the percentage of GFP-fluorescent cells detected in protoplasts suspension when transformed with increasing concentrations of plasmid **(E)**. Error bars represent the standard deviation.

Protoplasts were obtained from established and highly regenerative cultures of *V. vinifera* embryogenic calli of the varieties Crimson s. and Sugraone. The digestion of 1 gram of callus in an optimized digestion mixture followed by protoplasts isolation yielded up to 6 x 10^6^ cells, for which the vitality was higher than 99% –as assessed by FDA staining (**Figures 2A, C**). Cells were PEG-transformed with a plasmid containing a 35S::GFP expression cassette to evaluate the best transformation conditions and transformation efficiency (**Figures 2B, D**). In contrast to leaf protoplasts, which typically start to emit fluorescence 6-8 h after transformation, no fluorescence was observed in the samples from embryogenic calli earlier than 20 h after transformation. The level of fluorescence peaked at 26-30 h and remained constant until the 48 h-time point, although fluorescent cells were still visible at 72 h, when monitoring was stopped. A dose-response experiment was also carried out in Crimson s. to determine the optimal quantity of DNA to be used in PEG-mediated transfections. As protoplasts are generally reported to require a considerable amount of DNA to be transfected, 10 μg, 20 μg and 40 μg of plasmid were used (**Figure 2E**) corresponding to a DNA concentration in the transformation reactions of 2.5 pM, 5.0 pM and 7.5 pM, respectively. An optimal concentration could not be determined, as the percentage of fluorescent cells increased linearly (R^2^ = 0.9953) with increasing concentration of DNA in the range tested, with very few GFP-positive cells detected at 2.5 pM and up to 20% with 7.5 pM. These values, although smaller than the ones reported for other species (Woo et al., 2015; Bernard et al., 2019; Bertini et al., 2019), were taken into account when performing the subsequent RNPs transformations.

**Figure 3:**
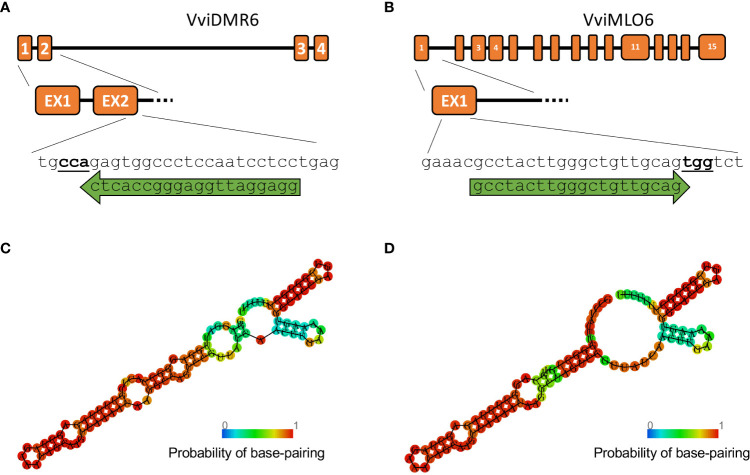
Guide RNA design. Gene structure of **(A)** *VviDMR6* and **(B)** *VviMLO6* with their respective guide RNA, indicated by green arrows. PAM sites are indicated in bold. A Minimum Free Energy (MFE) model was built for both guides **(C, D)** using the RNAfold online tool ((Gruber et al., 2008)) to verify their secondary structures.

**Figure 4:**
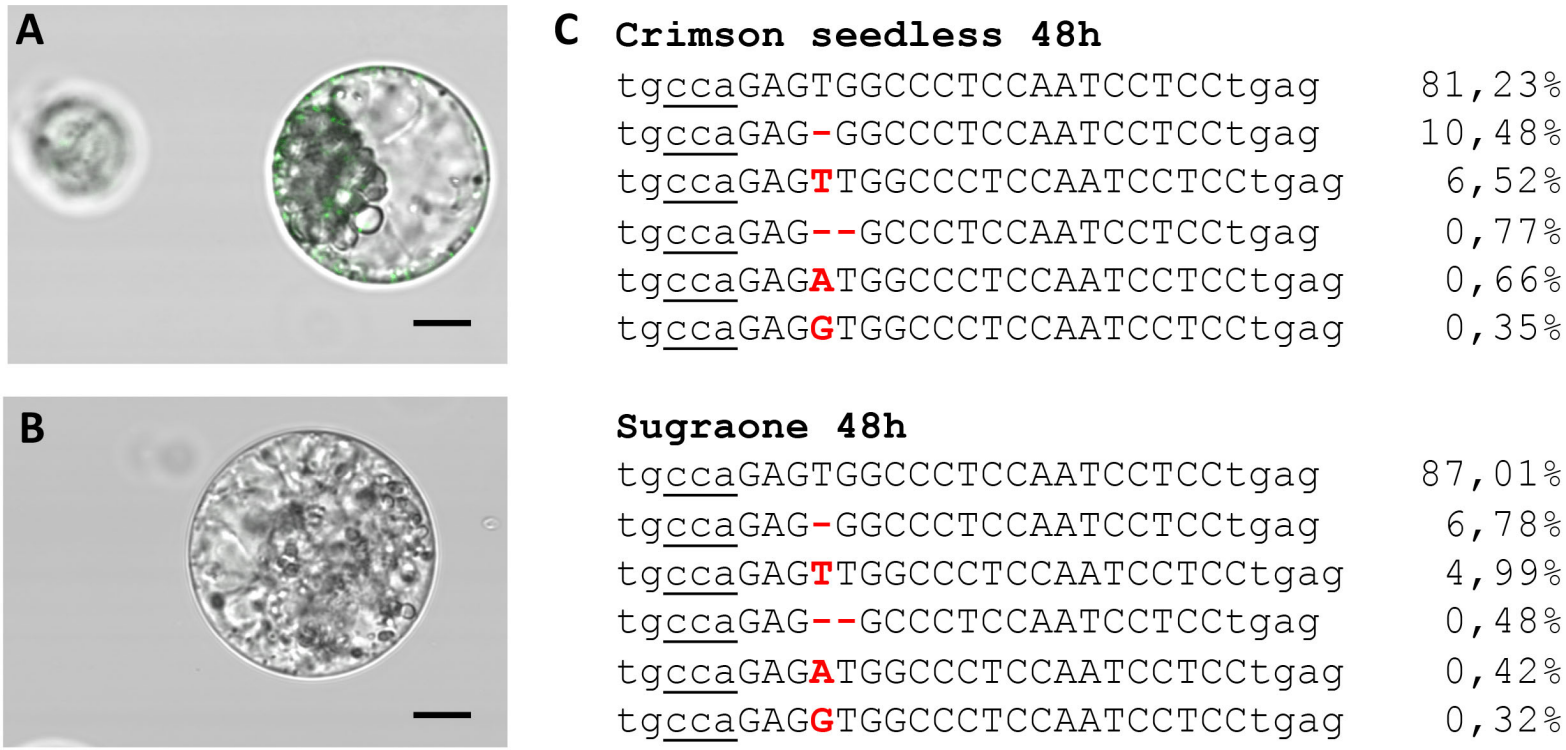
Transient transfection of protoplasts. **(A)** Image taken 2 h after transfection with Cas9-GFP RNPs (fluorescent endocytosis vesicles are visible) and **(B)** negative control. **(C)** Amplicon deep sequencing of DNA extracted from protoplast populations of Crimson s. and Sugraone at 48 h after transfection with RNPs with guide RNA targeting *VviDMR6*. The total amount of reads was 43828 and 53995, respectively, and the percentage of edited and wild-type (first raw in the alignment) reads is indicated.

The ability to deliver RNPs in grapevine protoplasts was explored with a GFP-tagged Cas9 protein (**Table 2**) and a guide RNA targeting *VviDMR6* (**Figure 3A**). Two hours after transfection, PEG-stimulated endocytosis vesicles were visible in almost all transfected cells (**Figure 4A**) but not in the control cells treated with PEG without the GFP-tagged Cas9 protein (**Figure 4B**), with a much higher efficiency with respect to pDNA (plasmid DNA) transformation. This could be explained either by the difference in molarity of the editing machinery in the protoplast suspension (370 nM RNP versus 7.5 pM DNA) and/or by the lower surface-charge of the RNP as compared to that of the DNA molecule. Total genomic DNA was isolated 24 h after transfection from the protoplast populations, but no editing on *VviDMR6* was detected by deep sequencing (data not shown). A similar experiment was performed by doubling the amount of RNPs, and with a Cas9 protein without fluorescent tag (**Table 2**): overall, 19% and 15% of the reads were edited in Crimson s. and Sugraone respectively, with an editing profile that was identical between the two varieties in terms of mutations obtained (**Figure 4C**).

### Protoplast transfection and regeneration

To perform the final knock-out experiments with RNPs, the guide-RNAs were designed (**Figures 3A, B**) to anneal to the N-terminus protein-coding region of the *VviDMR6* (Vitvi13g01119) and *VviMLO6* (Vitvi13g00579) genes, thus inducing frameshift-causing mutations. The choice, among all the possible guide RNA sequences available, was based on the secondary structure which ensured the most efficient interaction with Cas9 protein, as already described in literature (Kumlehn et al., 2018) (**Figures 3C, D**). Similarly to the transient RNPs assay previously described, 2.5 x 10^5^ viable protoplasts (i.e. positive to FDA staining, **Figure 1A**) were transfected, embedded in alginate discs (**Figure 1B**) and successively laid into a liquid growth medium (**Figure 1C**), whose osmolarity (510 mOsm) was reached with a mixture of sucrose and glucose. The quantity of glucose was progressively scaled down every two weeks, until zero at the 2^nd^ month of liquid culture. The discs containing microcolonies of proliferating cells (**Figure 1D**) were monitored until the appearance of embryos at the heart stage was visible (**Figure 1E**). Next, the discs were transferred into solid medium and in the dark. After few weeks, mature embryos could be observed (**Figures 1F, G**) and placed under the light (**Figure 1H**) to obtain the first shoot (**Figure 1I**). Plants obtained from those embryos (**Figure 1J**) were propagated in solid Nitsch medium (**Table 1**) and the genomic DNA from the leaves was extracted to assess editing in the target region. Overall, successful plant development, showing a normal growth phenotype upon acclimatization, was obtained within 6 months from the start of the experiment (**Figure 1K**). To assess the gene editing efficiency, Sanger sequencing was performed on PCR amplicons including the regions targeted by CRISPR/Cas9. In the case of biallelic homozygous editing, Sanger sequencing proved to be sufficient to assess the plant genotype, however deep sequencing of the target region was key to reveal any other allelic configuration.

In total 96 plants were regenerated and screened: 39 plants of Crimson s., and 57 of Sugraone, which appeared to be more regenerative. The editing experiment of *VviDMR6* in Crimson s. produced 8 plants, 5 of which were fully edited (homozygous and biallelic, with small indels ranging from -2 to +1 bp, **Table 3**). From the editing experiment with the MLO6 guide in Crimson s., a higher number of plants (31) were regenerated but only two were edited and at one allele (hemizygous) (**Table 3**).

**Table 3:** Allelic profile of the plants regenerated from protoplasts of Crimson seedless.

Genotype of Crimson s. plants regenerated from protoplasts.			
plant #	allelic profile	genotype description	targeted gene
1	-1/-1	biallelic, homozygous	*VviDMR6*
2	-2/-2	biallelic, homozygous	*VviDMR6*
3	-1/-1	biallelic, homozygous	*VviDMR6*
4	-2/-2	biallelic, homozygous	*VviDMR6*
5	+1/+1	biallelic, homozygous	*VviDMR6*
6	+1/wt	monoallelic	*VviMLO6*
7	-2/wt	monoallelic	*VviMLO6*

In the second column the number of nucleotides added or deleted by the mutation is indicated.

The *VviDMR6* editing experiment in Sugraone produced 15 plants with different mutation patterns out of 34 regenerated plants, while the *VviMLO6* editing experiment produced 7 edited out of 18 regenerated plants, all with the very same allelic and mutation profile (**Table 4**).

**Table 4:** Allelic profile of the plants regenerated from protoplasts for the cv. Sugraone.

Genotyping of Sugraone plants regenerated from protoplasts.			
plant #	allelic profile	genotype description	targeted gene
1	+1/+1	biallelic, homozygous	*VviDMR6*
2	-1/wt	monoallelic	*VviDMR6*
3	+1/wt	monoallelic	*VviDMR6*
4	-1/wt	monoallelic	*VviDMR6*
5	+1/wt/wt/wt	putative cell fusion	*VviDMR6*
6	-1/-1/ -1/wt	putative cell fusion	*VviDMR6*
7	-1/wt	monoallelic	*VviDMR6*
8	wt/-1/wt/wt	putative cell fusion	*VviDMR6*
9	-1/wt/wt/wt	putative cell fusion	*VviDMR6*
10	-1/wt	monoallelic	*VviDMR6*
11	-1/-1/ +1/+1	putative cell fusion	*VviDMR6*
12	-1/+1/ wt/wt	putative cell fusion	*VviDMR6*
13	+1/wt	monoallelic	*VviDMR6*
14	+1/wt	monoallelic	*VviDMR6*
15	-6/wt	monoallelic	*VviDMR6*
16	+1/wt	monoallelic	*VviMLO6*
17	+1/wt	monoallelic	*VviMLO6*
18	+1/wt	monoallelic	*VviMLO6*
19	+1/wt	putative cell fusion	*VviMLO6*
20	+1/wt	monoallelic	*VviMLO6*
21	+1/wt	monoallelic	*VviMLO6*
22	+1/wt	monoallelic	*VviMLO6*
23	+1/wt +1/wt	both monoallelic	*VviDMR6 + VviMLO6*
24	-1/wt +1/wt	both monoallelic	*VviDMR6 + VviMLO6*

In the second column the number of nucleotides added or deleted by the mutation is indicated.

In a diploid organism with uniform genetic background (i.e., no chimerism), the allelic ratio can be easily revealed by deep sequencing. Only one type of read is observed in case of homozygosity and two types of reads with a 1:1 ratio in the case of heterozygosity/hemizygosity. Indeed, this was the case in our experiments, where the percentage of mutated reads was either 50% or 100% (or 0% in non-edited plants), validating the hypothesis that -being derived from single cells- these plants cannot be chimeric. The only exceptions were found in Sugraone, especially in the *VviDMR6* editing experiment, where 5 out of 16 plants showed more than two alleles upon sequencing. In these cases, the four alleles were always represented by exactly 25% of the reads, suggesting an event of cell fusion of wt and edited protoplasts likely occurring during the PEG-mediated transfection (Reinert and Yeoman, 1982) (**Supplementary Figure 1**). These plants showed a more stunted growth compared to the others, probably because of the abnormal ploidy level or DNA quantity.

Possibly, undesired cell fusion could be avoided by reducing either PEG concentration or the duration of PEG treatment or by alternative methods, such as electroporation or mechanoporation (Sharei et al., 2013; Chakrabarty et al., 2022).

The multiple occurrence, in independent lines, of the same type of mutation like the one here reported in case of *VviMLO6* in the cv. Sugraone, supports the findings that DNA repair after non-homologous end-joining (NHEJ) is not a random process, but it depends on some features of the Cas9-targeted locus (van Overbeek et al., 2016). Considering the higher regeneration ability of Sugraone protoplasts and the more predictable and consistent efficiency of transformation, we explored the possibility of duplex gene-editing, by using, in a small-scale experiment, both guide RNAs for *VviDMR6* and *VviMLO6*. In this case, out of 5 plants regenerated, 2 were edited in both genes (with monoallelic mutations) with no signs of cell fusion or chimerism (**Table 4**). Obtaining a complete knock-out in more than one target gene likely requires the screening of a larger number of regenerants.

**Figure 5:**
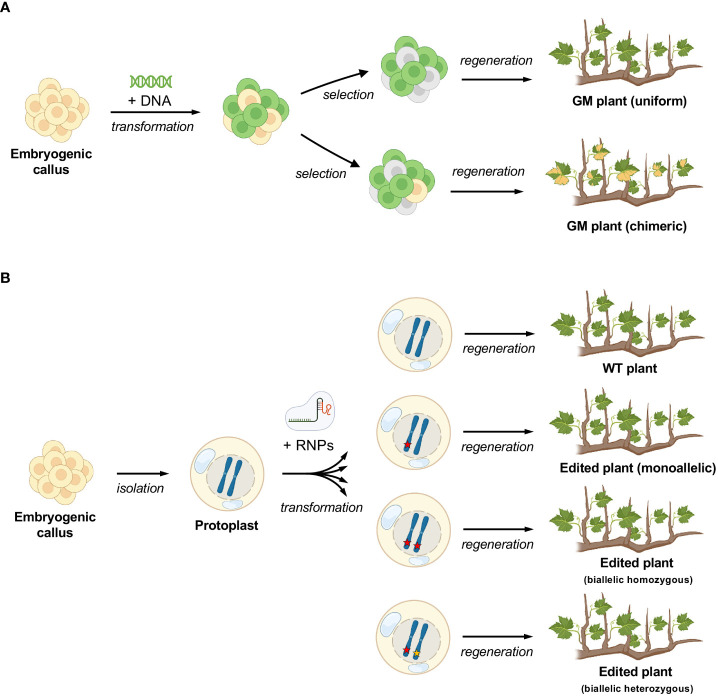
Differences in the process of regeneration of grapevine plants between callus and protoplasts. **(A)** standard process with exogenous DNA insertion and possibility of chimerism (depicted here by variegated leaves) **(B)** protoplast regeneration, where there is no possibility for chimerism. The different editing outcomes are simulated (editing events are depicted by red and yellow stars).

Chimerism is a rather frequent problem encountered in the transformation of grapevine mediated by *A. tumefaciens* (Ren et al., 2016; Dalla Costa et al., 2017; Nakajima et al., 2017) (**Figure 5A**). To reduce the chances of obtaining chimeras, transformed callus can be propagated prior to induction to embryos, in a selective medium. However, this procedure accumulates somatic mutations and reduce regeneration efficiency (Nakajima et al., 2017) and therefore it is generally avoided. The use of CRISPR/Cas9 provides an additional level of complexity in the generation of chimeras, which strictly depends on the cutting efficiency of the editing machinery and on the outcome of the endogenous repairing process. Chimerism can lead to only partial expression of the desired trait throughout the transformed plant (i.e. some parts of the plant are mutated, others are not) or in the best case to the lack of uniformity in the genotype (i.e. the whole plant is mutated but different parts have a different type of mutation). The issue of chimerism has been nowadays highlighted by NGS, required to detect editing, but largely unnecessary prior to the gene-editing era, when most of genetically modified grapes were overexpressor or silencing mutants. Once present, the elimination of chimerism in grapevine is theoretically possible by *de-novo* embryogenesis or organogenesis followed by a second step of genetic screenings to select non-chimeric plants from the new regenerants, but such a process would be very time-consuming. Regeneration from single cell has the advantage to abolish or strongly reduce chimerism from the start, as reported in this study for grapevine and previously in other species (Cankar et al., 2021) (**Figure 5B**).

## Conclusions

Despite the advances recently achieved with CRISPR/Cas technology have led to numerous applications in plant biology, the regeneration step still embodies a critical point in the workflow leading from single-cell editing to the plant phenotyping, in particular in the case of woody plants including grapevine. In this respect, this work provides a robust and rather efficient methodology to regenerate edited grapevine plants through a DNA-free and single-cell based CRISPR/Cas technology. Very recently Najafi et al. has also shown editing and protoplast regeneration in a GFP-expressing line of the table grape Thompson seedless with similar efficiency [(Najafi et al., 2022)]. The methodology here outlined could serve a larger and more general interest, being potentially extended to either base- or prime-editing applications (Ren et al., 2022), thus going beyond the mere gene knockout.

## Data availability statement

The original contributions presented in the study are publicly available. This data can be found here: NCBI, PRJNA899691.

## Author contributions

SS, CM, MM and JV conceived the research SS designed the experiments. SS and US performed the experiments and collected the samples. LG provided embryogenic callus. LG and TZ designed the sgRNA. The manuscript was written by SS and US and revised by SS, US, LG, TZ, MM, JV and CM. All authors contributed to the article and approved the submitted version.

## Funding

This work was supported by Fondazione Caritro, Bando Ricerca e Sviluppo 2018, which has granted the project TRADING (Transfer of molecular probes into plant cells for genome editing and transcriptional profiling). Further financial support was provided by FondazioneVRT (Trento) which has granted the project Vitis-SCT (call 3°-Bando Impact Innovation 2021).

## Acknowledgments

Some figures were created with Biorender (https://biorender.com). A previous version of the manuscript was pre-printed on BioRxiv and can be found at the following link: https://www.biorxiv.org/content/10.1101/2021.07.16.452503v1.

## Conflict of interest

SS, US, LG and TZ are named inventors on a patent application pertaining to the technology filed by Fondazione Edmund Mach and Enza Zaden. US is also an employee of Consorzio Innovazione Vite (CIVIT).

The remaining authors declare that the research was conducted in the absence of any commercial or financial relationships that could be constructed as a potential conflict of interest.

## Publisher’s note

All claims expressed in this article are solely those of the authors and do not necessarily represent those of their affiliated organizations, or those of the publisher, the editors and the reviewers. Any product that may be evaluated in this article, or claim that may be made by its manufacturer, is not guaranteed or endorsed by the publisher.
