# Supplementary material for: Regeneration of non-chimeric plants from DNA-free edited grapevine protoplasts
Source: Front Plant Sci. 2022 Dec 1;13:1078931. doi: 10.3389/fpls.2022.1078931 (PMC9752144; doi:10.3389/fpls.2022.1078931)
Supplement: Supplementary file 1 [file DataSheet_1.pdf]

Supplementary TABLE 1. Step-by-step version of the protocol described in the Material and Method section. The media/buffer compositions and the reagents used are described in Table1 and Table 2.

Step	Description	Approx. Time	Method Section in the main text
Protoplast preparation	Prepare the enzymatic mixture and place 1 g of embryogenic callus in 13 ml of enzymatic mixture	0.5 h	3.1
	Mix on a tilt shaker at 25°C for 16 hours in the dark	16 h	
Protoplast purification	Filter through a 60 μm nylon sieve and collect (80 g x 4’, no brake)	15 min	
	Wash in MMG and layer on a 16 % w/v sucrose cushion by centrifugation (90 g x 4’, no brake)	20 min	
FDA stain	Stain with FDA (50x stock solution in acetone) for 5 minutes to check for vitality	10 min	
Transfection	Dilute cells at 10^6/ml and take an aliquot of 250 μl for each transfection	10 min	3.2
	Mix 40 μg of Cas9, 40 μg of sgRNA and 10 μl of NEBuffer 3 (or in alternative 20 μg of plasmid) with an aliquot of cells and 250 μl of PEG-calcium solution in a falcon tube. Pipette gently and incubate for 10’ at room temperature	20 min	
	Wash with WI buffer by adding 10 ml and collecting the cells at 80g x 4’. Resuspend in 250 μl WI buffer	15 min	
Agarose embedding	Mix equal volumes of cell suspension in WI buffer with cultivation medium in 1% (w/v) Low Melting Agarose PPC close to room temperature. Dispense 50-100 μl droplets into a Petri dish and wait for gelification. The droplets can be soaked in the same liquid medium and conserved for the following days for the microscopy imaging.	1 h	3.2
Alginate embedding	Adjust the protoplasts density to 2×10^5 cells/ml with WI buffer, then add an equal volume of alginate solution and gently mix. Cast 1 ml of the resulting suspension on calcium-agar plates, leave 1h to solidify	1.5 h	3.4
Protoplast culture for regeneration	Move the disks into new Petri with Nitsch-based liquid medium. Place at 24 °C in darkness. Change weekly with fresh medium.	1 h	3.4
	Change medium using 75% of the initial glucose concentration (225mM final)	Week 2	
	Change medium using 50% of the initial glucose concentration (150mM final)	Week 4	

	Change medium using 25% of the initial glucose concentration (75mM final)	Week 6	
	Transfer the disks on solid GS1CA enriched with 300 μM glutathione. Cut the disk into smaller pieces in the process.	Week 8	
Plant obtainment	Move embryos in Nitsch and Nitsch solid medium at 16/8 light/dark photoperiod (80-100 μmol m^{-2} s^{-1}) at 24 °C.	Week 12-16	3.4

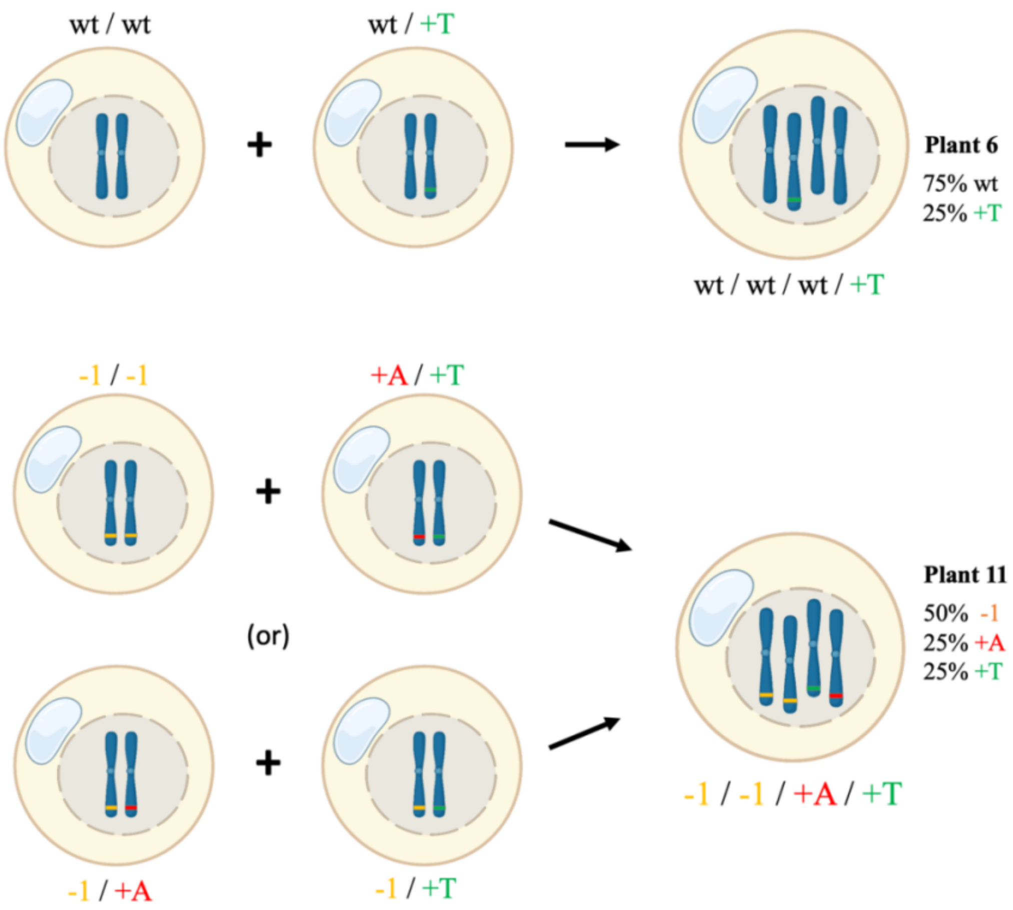

Supplementary Figure 1. Schematic model of cell fusion and change of ploidy as inferred by amplicon deep sequencing in this study. (Upper part) Explanation of the 75%/25% reads ratio found in plants 5, 6, 8 and 9. (Lower part) Explanation of the 50%/25%/25% reads ratio found in plant 12.
